# How do motorcyclists manage mental tensions of risky riding?

**DOI:** 10.1186/1471-2458-13-865

**Published:** 2013-09-20

**Authors:** Shahrzad Bazargan-Hejazi, Fereshteh Zamani-Alavijeh, David Hindman, Esa Mohamadi, Mohsen Bazargan

**Affiliations:** 1 Department of Psychiatry, Charles R. Drew University of Medicine and Science, & David Geffen School of Medicine, University of California, Los Angeles, CA, USA; 2 Department of Public Health, Social Determinants of Health Research Center, Faculty of Health, Ahvaz Jundishapur University of Medical Sciences, Ahvaz, Iran; 3 Department of Family Medicine, Charles R. Drew University of Medicine and Science, Los Angeles, CA 90059, USA; 4 Department of Nursing, Tarbiat Modarres University, Tehran, Iran

## Abstract

**Background:**

Road traffic injuries, especially those involving motorcycles, are a particular concern in Iran. We aimed to identify the specific cognitive dissonances and consonances associated with risky riding among Iranian motorcyclists.

**Methods:**

This was a grounded theory qualitative study of male motorcyclists who were ≥18 and were living in one of the three cities of Tehran, Isfahan and Ahwaz. Thirty four (n = 34) motorcyclists participated in 19 in-depth interviews and 5 focus-groups between January 2007 and February 2008.

**Results:**

We identified four categories of motorcycle riders each endorsing a unique risk bias they employed to justify their risky ridings. The categories included: (1) Risk Managers who justified risky riding by doubting that it would result in negative outcomes if they are competent riders. (2) Risk Utilizers who justified risky riding as functional and practical that would enable them to handle daily chores and responsibilities more efficiently. (3) Risk Calculators who justified risky riding by believing that it will help them to avoid road crashes. (4) Risk Takers who justified risky riding by arguing that risky riding is thrilling and brings them peer recognition.

**Conclusion:**

Our findings reveal different groups of motorcyclists according to their different rationalizations for risky riding. Road safety advocates can benefit from our findings by matching relevant and appropriate interventions and incentives to these specific groups.

## Background

The World Health Organization (WHO) identifies motorcyclists as one of the most vulnerable groups for road traffic injuries (RTIs) [1]. Motorcyclists and their passengers are 37 times more likely to die in traffic injury than riders in four wheeled vehicles [1,2]. Motorcyclists also are at high risk for head trauma [3-5]. In addition, compared to helmeted riders, unhelmeted riders are 40% more likely to suffer a fatal head injury and 15% more likely to suffer a nonfatal injury when involved in road traffic crashes [2].

Road traffic injuries, especially those involving motorcycles, are a significant concern in Iran where RTIs are the leading cause of unintentional injuries [6-8] with approximately 70 estimated deaths per day [9,10]. In the capital city of Tehran alone there were more than 2 million registered motorcycles in 2003 [11]. In 2008 motorized 2- and 3-wheelers made up 37% of the country’s 17 000 000 registered vehicles, commonly used by men [12], and in 2010, there were more than 8 million monocycles in Iran [13].

Empirical data shows that motorcyclists in Iran have less favorable attitude toward safety riding measures and are more likely to normalize risky riding [12,14]. Government reports estimate an upward trend in the number of registered vehicles in Iran, and warn of their high contributions in RTIs and road traffic fatalities [15]. Despite ample evidence establishing risks factors for RTIs among motorcycle riders in Iran [14,16,17], little is known about motorcyclists’ specific thought processes and risk biases with regard to risky riding.

Research in other countries suggests that public awareness of motorcycles as one of the least safe modes of transportation is high [18,19]. However, an increasing number of road user safety research studies have concluded that motorcyclists have a different perception of safe-riding or accept different levels of risk from the rest of general public [18,20]. Wilde believes that an individual’s perception of risk determines, consciously or subconsciously, their targeted accepted level of risk [21]. It is suggested that among their own group, motorcyclists’ motivations towards risk vary from high ‘sensation seekers’, to ‘risk averse’, ‘risk acceptors’, and ‘risk seekers’ [22]. Christmas et al., have categorized motorcyclists into seven categories to reflect their different motivations for riding, related to passion, performance and practicality, which also differed in attitudes and perceptions towards risky riding, as well as their acceptance of safe riding measures. The seven categories include: Riding Disciples, Riding Hobbyists, Performance Disciples, Performance Hobbyists, Car Aspirants, Car Rejecters, and Look-at-me Enthusiasts [23].

One common aspect shared by the majority of motorcyclists in the aforementioned findings is that they are aware of road traffic related risks and injuries associated with motorcycle riding. Assuming this awareness is also high in Iran, yet Iranian motorcyclists have a high acceptance of road risk [14], this raises the question as to whether there is a disconnect between their awareness of risk and risk behavior. Cognitive dissonance theory provides an appropriate theoretical context in which to explore this question [24]. According to this theory, people should experience mental tension when their beliefs and actions are inconsistent. Mental tension, also referred to as cognitive dissonance, may include regret, guilt, discomfort, etc. and depending on the magnitude of the tension, the individual may engage in a cognitive process to minimize the dissonance they experience. More specifically, we set out to explore how Iranian motorcyclists cognitively process the unintended conflict between their actions (e.g. risky motorcycle riding) and the awareness of the inherent risks involved in motorcycle riding (e.g. the increased risks death, injury, etc.).

## Methods

### Study design

We chose grounded theory method to conduct and collect data for this study. Grounded theory provides appropriate context to uncover the process of engagement in a behavior from the participants’ perspective [25].

### Settings and participants

The study was conducted in three major cities in Iran; Tehran (The Capital), Isfahan and Ahvaz, between January 2007 and February 2008. Potential participants were identified by the study principle investigator (FZA) by approaching motorcyclists in the street and at motorcycle parking areas in the cities. Only males were approached as motorcycling is almost entirely a male activity in Iran. At the time of recruitment, motorcyclists were asked: 1) if they were 18 years of age or older; 2) if they were living in one of the three cities of Ahwaz, Tehran, or Isfahan at the time of the study, and 3) if they consider their riding a risky behavior. ‘Risky behavior’ was defined as a behavior that would increase the likelihood of injury or death to them or others. If meeting eligibility criteria including self-identifying as a risky rider, they were invited to choose to attend either a focus group or interview at the researchers’ offices at a later date, when written informed consent was obtained before participating in the study. Ahvaz, Jundishapur University of Medical Sciences Ethics Committee approved the study protocol. Twenty-Six male motorcyclists participated in five focus group discussions, and eight motorcyclists participated in one-on-one in-depth interviews, therefore a total of N = 34. The breakdown of participant numbers by cities is included in Table 1. The mean age, standard deviation, and age range of participants were 32 ± 13 and 19 to 51, respectively.

**Table 1:** Numbers of participants in focus groups and in-depth interviews

**City**	**Focus group (Participants)**	**In-depth interview (Participants)**
Ahvaz	3 (15)	5 (4)
Tehran	1 (6)	2 (2)
Isfahan	1 (5)	2 (2)
**Total**	5 (26)	9 (8)

### Data collection

We used a two-pronged data collection method which included in-depth interviews and focus-group discussions. Data collection was directed by the principles of convenient purposive sampling and data saturation [26]. Convenient purposive sampling is a nonprobability sampling method that is used when researchers are seeking participants with specific characteristics that set them apart from the others. Data saturation refers to a point of ‘diminishing return’ to a qualitative sample. Specifically, it is a point in data collection process during the course of a study that no new or relevant information emerges, or when the addition of more data does not add to the already collected information. These are the suggested methods in qualitative studies that allow for richer analytic generalizations [27]. Data collection and analysis were initiated in Ahvaz and continued until we reached data saturation. Subsequently, we continued with purposive sampling in Tehran (the Capital) and Isfahan (another major city in Iran) to confirm data saturation.

The study principal investigator (FZA) moderated the focus group sessions and along with the study co-investigator (EM) conducted the in-depth interviews. To align study questions with study aims we used the following open-ended questions in the in-depth interviews and focus group discussions: *1) what is your understanding of the risks involved with motorcycling? 2) What causes those risks? 3) Do you feel susceptible to those risks? 4) If yes, how do you manage or cope with the mental conflict/tension created by risky driving? In other words, how do you reconcile your actions with your perception of risks*? These questions were followed by probing questions in order to further engage participants to provide more thoughtful and detailed answers. The last two questions were the key questions for this study. Each in-depth interview lasted 30–40 minutes while the focus-group discussions each lasted 50–90 minutes.

### Data analysis

Recorded discussions and interviews were transcribed verbatim. Subsequently, we performed content analysis to identify meaningful units/phrases (manifest content) in the data. This process was performed using Wilms et al., methodology of “Coding Consensus, Co-occurrence, and Comparison” that is rooted in grounded theory [28]. This methodology entailed reviewers independently reading and rereading the initial verbatim transcript and identifying key sub-themes according to the participants’ phrases (latent content) and performing initial coding. Initial codes then were compared and discussed among the reviewers and were categorized according to their similarities and differences [29-32]. Identified themes were then condensed further into broad themes. All subsequent transcripts were then coded utilizing this master codebook. Finally, the relationships among the broad themes were considered with a focus on their utility in operationalizing salient domains across individual interviews and focus groups. In addition to the aforementioned process, two experts outside of the study team, as recommended in the literature, randomly selected sections of the transcripts and independently followed the aforementioned process to subsequently verify and resolve identified coding and thematic issues. The study team also met on a regular basis to ensure that the qualitative data analysis was systematic and verifiable, as recommended by the experts [32-34].

### Trustworthiness - validity and reliability

Several provisions were made by the researchers to enhance the credibility of the data [33]. First triangulation of data, i.e., use of both one-on-one in-depth interviews and focus groups compensated for each method’s individual limitations. Second, scrutiny and evaluation of the transcripts, codes and categories by bilingual members of our research team as well as colleagues outside of the research group ensured that the nuances of data were reflected in coding and added more feedback and fresh perspectives to the data. Third, the qualification and expertise of our research team, as well as their multidisciplinary background were invaluable in collecting rich data and conducting reliable analysis [29,35,36].

The research team consisted of members with different background and expertise all relevant to implementation of current study. The team included a public health specialist from Iran who also was the principal investigator of the project (FZA), a nurse with a background in qualitative research (EM), two medical sociologists (SHB and MB), and a clinical psychologist (DH) from the U.S.A. Members of the research team were all experienced and active in conducting qualitative and quantitative research.

## Results

The thirty four (n =34) participants in this study were all male motorcyclists who were self-identified as risky riders and were aware of the health-related consequences of risky riding. The responses of the participants regarding how they perceived risky riding and how they attempted to resolve and minimize any resulted mental tension allowed us to categorize them in four different groups of motorcyclists. This included: 1) Risk Managers; 2) Risk Utilizers; 3) Risk Calculators; and 4) Risk Takers.

### Risk mangers

These motorcyclists admitted that they were aware of the negative consequences of risky riding. They agreed that they were vulnerable to these risks but they also expressed a belief they were capable of managing the risks associated with motorcycling. They commented that they were able to recognize when and how to be cautious while riding, that they could recognize risks when they presented and that they dealt with them as they came. These motorcyclists were confident that they were immune/invulnerable against any crashes or injuries and believed that engaging in risky behavior was a rite of passage for skilled riders. They commented that their previous experiences played a role in them having a high sense of self-assurance. To these riders, risky riding was a means to becoming a competent rider. The following phrases indicate their attitudes and understanding of risks involved in risky riding:

“*I ride quite fast and don’t follow most of the traffic rules and regulations. I know when to yield to other drivers, use the sidewalk for shortcuts, when to stop and when to speed (37 years old).”* “*I can estimate at what speed cars are approaching the intersections and when they [will] get to the intersection. Therefore, I can make an educated guess whether or not running the red light can cause an accident or not (27 years old).”* “*When I want to ride on the sidewalks or [the wrong way on a] one-way street, I make sure I take all the necessary measures to avoid accidents (30 years old motorcyclist).”*

### Risk utilizers

Of the motorcyclists in the study, those who belonged to the Risk Utilizers category had a defined notion that risk-free riding is not practical due to existing driving and road traffic circumstances. However, these riders believed that having a motorcycle was very practical, handy and useful for managing daily life necessities. They did not deny the dangers of risky riding, but tended to minimize the negative consequences of risky riding and instead stressed its benefits and utility for managing daily chores and responsibilities while saving time. They considered risky riding a ‘means’ to achieve quick mobility and increased functionality as reflected in the following excerpts:

“*I agree that riding a motorcycle is more dangerous than a car. After all, it only has two wheels and at any time one may lose balance. But still, it is worth it. I easily ride it on the sidewalks or anywhere else for that matter, and take care of my daily tasks much quicker than riding in cars (35 years old).” “I have to spend all my time either in traffic or behind red lights without getting much done. I am completely handicapped without my motorcycle (28 years old motorcyclist).”*

### Risk calculators

These riders were willing to take calculated risks. They attributed their previous road crash experiences to their miscalculation of risks. They believed that road crashes were avoidable if one paid good attention to the road and passenger cars, and remembered previous mistakes that caused a road crash. The younger participants in particular were more persuaded by this belief. They articulated that their previous crashes were good reminders of their miscalculation in avoiding crashes. Otherwise, they were not likely to question their risky riding behaviors. For example, they disagreed with the notion that riding over the speed limit might have caused their recent crash. For this group, risky riding was perceived as a means to avoid crash possibilities and stay safe. The following quotes reflect the comments of these participants:

“The other day, if I was smart enough, I would have calculated the distance between my motorcycle and the approaching car in the intersection correctly. Indeed, I could have escaped the crash if I had done so while running the red light (25 years old motorcyclist).” “I always ride on the sidewalk and nothing happens; the other day I was riding on the same path but it happened that they had washed the sidewalk, so, I lost control of my motorcycle and crashed (25 years old motorcyclist).”

#### Risk takers

The attitude of these riders was that motorcycling was exciting and daring. These riders expected motorcycling to be risky and they were attracted to these risks. They actively promoted the excitement and thrill of risky riding amongst their peers and challenged them to perform risky stunts. Even though they reported having a few close calls or road crashes over the years, it appeared that their passion for risky riding overshadowed their concerns from previous crashes. These riders engaged in difficult stunts to show off their talent and skills. They also did not hesitate to combine risky riding with drinking to attract the interest of their peers. These motorcyclists considered risky riding a means to achieve pleasure and gain the attention and admiration of others. Younger riders, for the most part, belonged to this group. The following quotes resonates this group of participants:

“*Riding a motorcycle with a helmet is no fun. The excitement is when you ride it without a helmet and feel the wind blow through your hair. Experiencing such a feeling is worth the risk. It is a feeling that driving a car cannot replace it* (24 years).*”* “*There is nothing like the feeling I get when I am on my motorcycle and ride it with full speed. It is an exhilarating feeling that nothing can replace. I will never give it up* (19 years)*.*”

## Discussion

Riders in our study attempted the following approaches to minimize the tension between their beliefs of the dangers associated with risky riding and reported continued risky riding behaviors: they 1) reduced the anticipation of experiencing negative outcomes by emphasizing their riding skills; 2) replaced worries about risky riding by emphasizing the practicality of the motorcycle; 3) changed the meaning of the risk; favoring calculative risk and defensive riding; 4) focused on the thrill of riding that minimized their assessment of the associated risks. This follows from Festinger’s claim that once individuals experience dissonance between their beliefs and actions they are motivated to reduce it in order to alleviate any resulting mental tension. To do so, he suggests people attempt to reduce the unpleasantness of their thoughts by: 1) changing either their thoughts or actions to relieve the tension (accommodation); 2) adding a new comforting thought that replaces the negative/conflictual thoughts with more positive ones; 3) changing the significance of either the thought or behavior through introduction of another mediating thought that decreases the conflict (assimilation), or 4) disregarding the significance of the unpleasant thoughts altogether [24].

### Reducing the anticipation of experiencing negative outcomes by emphasizing one’s riding skills

Of the categories we identified in the study, the Risk Mangers seemed biased toward their own riding skills. These riders argued that as competent riders they can push their limit in the road and anticipate and manage road-related risks. Similarly, in an Australian study, Watson et al., [37] reports that motorcyclists in their study believed that competent riders can challenge the set limits in the road and still be considered safe riders. Our riders viewed every positive experience or escape from a dangerous situation as a testimony of their skilled riding and capabilities that would set them apart from novice riders. They believed these experiences were yet another reason to believe that they could avoid the future crashes. A similar view that skillful riders can avoid road crashes has been reported by motorcyclists in other studies [18,20,38]. These riders believed in defensive riding and did not feel compelled to change their riding habits.

Whether or not these motorcyclists are experiencing an “illusory sense of control” is a question our data is unable to answer and requires further study. However, some investigators claim that such seems to be the case in risky drivers who overestimate their driving skills which ultimately contributes to driving violations and crashes [39-42]. Wilde asserted that our past experience with risky situations determines our assessment of future potential risks. As such, every time we survive a risky situation we raise our target level of risk [43]. Hence, our findings suggest that road safety promoters should be cognizant of “positive self-bias” and high level of risk tolerance in this group. In this respect, we tend to believe that information campaigns that focus on negative outcomes of risky riding would not appeal to this group since they may view it inaccurate, irrelevant and in conflict with their personal experiences. This requires further investigations.

Similar to the Riding Disciples in Christmas, et al., categorization, these riders seem to believe avoiding risk is a personal responsibility and did not pursue risk for its excitement alone [23]. Therefore, they may be more attentive and attracted to road-related safety campaigns that enhance their inner needs for achieving riding competency and control. Future studies are needed to inform us if such campaigns are effective in changing motorcyclists’ behaviors. In their view, a competent rider can manage the anticipated road-related risks, avoid crashes, minimize injury, and be relatively safe. Jderu reports that experienced motorcyclists have a tendency to view younger novice riders as “out-group” (i.e., outsider group) who expose themselves and others to far more risk than more experienced riders [44]. In this respect road safety interventions may benefit from active engagement of more competent riders in delivering road safety education and training to novice riders. The unintended consequence of this could be that this group may keep their own riding habits in check. Further empirical investigation could provide evidence as to whether these types of interventions address the issue.

### Replaced worries about risky riding by emphasizing the practicality of the motorcycle

Risk Utilizers used risky riding to their advantage in order to manage daily work and responsibilities. These riders found motorcycle to be practical for everyday use and quick access to work. They also claimed motorcyclists must be aware of the road-related risks and must learn how to handle them. In this respect, they resembled the Risk Manager category. But unlike this group, they were not motivated by an innate need for competence and autonomy. Instead, they justified risky riding for its functionality. Therefore, safety interventions that focus on functionality of safe riding practice and safety gear could be enticing to these riders. Further empirical data are needed to support this notion. Christmas et al., reported that ‘use of safety gear’ was rated highly by the Riding Disciples in their study whose primary purpose in riding was to arrive safely [23]. Similar to these riders, Risk Utilizers in our study did not seem to welcome risk and they were motivated to reach their destination safely because their work depended on it. Future interventional studies can assess if safety tips that enable these riders to function more effectively and safely are attractive to these riders.

### Changed the meaning of risk; favoring calculative risk

The Risk Calculators in our study more commonly used this approach to justify risky riding. They were comfortable with taking risks as far as it was calculative and aimed to minimize road crashes and injuries. They were biased by the notion that risky riding is the only way to deal with dangerous road situations and avoid road crashes. These riders minimized mental tension associated with risky riding by maximizing crash protecting propensity of their riding style. In contrast with typical risk takers who are less likely preoccupied with traffic-related crashes, riders in this category were preoccupied with crash incidences and application of crash aversive behaviors. Similar to the Risk Managers in our study and the Riding Disciples in Christmas et al., study, the attitude of these riders was to manage risks [23]. But unlike the Risk Mangers whose risky riding was perceived as making them more competent, the Risk Calculators described their risky riding behaviors as protecting them from risky road situations.

This group also resembled the Performance Disciples in Christmas et al., study by holding a precautionary fatalistic attitude about risk and a willingness to live with perceived inherent risks. A similar view was expressed by the motorcyclists in Musselwhite’s study [18,23]. They perceived the road as a competitive space that forces riders to behave aggressively and take calculative risks. It is questionable whether this group of riders favors the use of safety gear, similar to the Riding and Performance Disciples in Christmas study [23]. Previous findings regarding Iranian motorcyclists suggest that, for the most part, they have a negative attitude towards use of safety helmet, considering it limiting when they need to vigilantly scape crash scene [14]. Christmas et al., argue that these types of riders are less likely to change their risky riding even if exposed to crash scenes that involve other motorcyclists. Therefore, fearful messages do not seem to motivate this group to reduce risk riding [23]. These riders have a preconceived notion that following traffic rules does not necessarily save them from crash incidence. The challenge for the road safety promoter is to influence risky riding behaviors of these types of riders by identifying effective enforcement incentives that would prevent them from making irrevocable riding decisions with regretful outcomes through both positive and negative reinforcement strategies (i.e. rewarding safe riding behaviors and implementing significant, consistent penalties for risky riding behaviors) [45,46]. In this respect, a synergistic-based approach can encourage cooperation among motorcycle safety advocates, insurance industry, and judicial and law enforcement organizations in developing, implementing, and monitoring of such incentives [2,8,47,48].

### Focusing on thrill of riding and having care free attitude towards risk

Of the categories in this study, Risk Takers showed the most relaxed attitudes towards risky riding and over-emphasized the thrills and excitement they experience by motorcycling. They seem to resemble Performance Hobbyists, and Look-at-me Enthusiasts in Christmas et al., study who were young and equally attracted to thrill of riding [23]. However, unlike Performance Hobbyists, Risk Takers in our study expressed a higher level of confidence in their riding, and unlike Look-at-me Enthusiasts, they admitted to taking risks. Moreover, like the Performance Disciples in Christmas et al., groupings, these riders were motivated to show off their riding abilities and speed. Christmas et al., report that young risky riders in their study viewed life without risk as boring and were biased towards considering themselves good riders and less vulnerable to risk [23]. Other researchers stress that young people seems to identify with their risk taking behaviors [49], which bring them a sense of higher self-esteem [50], as those behaviors are reinforced [51] by their peers as fun [52]. Allen and Brown assert that acting against the norms of safety establish young drivers’ credibility and status among their peers [53]. The Risk Takers in our study also justified their thrill seeking riding as an activity that was fun and attracts the admiration of their peers. In that respect, they showed willingness to compromise safe-riding practices to obtain the approval of their peers. They seem to resolve mental tension (if any) associated with risky riding by arguing that risky riding is socially valued among their peers and, therefore, justified. Moreover, this group appeared more resistant to changing their riding behaviors. Previous studies have also reported that younger motorcyclists have lower perceived risk of experiencing a road crash [54], and are highly motivated by the immediate gratification associated with risk taking and sensation seeking, when compared to drivers [22,37,55].

Of the approximately 75 million people living in Iran, approximately 24 million (32%) are 20 to 34 years old [56], and by popular belief seeking enjoyment and excitement is viewed as a normal part of being young [57]. Considering the documented greater risk taking behaviors in younger motorcyclists [16], their perceived invulnerability, misperception of risks, and unwillingness to use protective gear [58,59], as well their bias regarding risky riding identified in this study, there is a particular need for continual attention to the risk-taking behaviors of young motorcyclists in Iran. This group of motorcyclists may benefit from interventions that address the impulsivity and emotional aspect of their riding [18]. Further qualitative studies can inform the development of interventions that include an alternative ‘thrill’ to replace the one young riders experience from risky riding. In addition, social marketing experts may be able to assist road safety advocates to identify and promote alternative ‘thrills’, and effectively communicate possible consequences of risky riding that could facilitate changing current cultural norms that discourage safe riding among this group. Further studies are needed to implement and test the effectiveness of such interventions .

While the aforementioned categories are not mutually exclusive, we discuss them separately to improve our understanding of the motorcyclists’ risk biases; how they perceive, appraise, and justify risks related to their own riding. Further, our findings may benefit road safety promoters and injury prevention specialists in developing targeted messages, incentives, or traffic enforcement measures that match with specific risk biases of motorcyclists in Iran.

### Limitations

While a qualitative approach may limit the reproducibility of our results, we were not aiming to identify a list of reliable and significant factors that could predict variations in behavior, as is the case in quantitative approaches. Instead, our goal was to gain an in-depth understanding of the cognitive contexts and rationalizations behind these variations in behavior.

As such, in the current study we were able to engage our study participants in a collection of data that revealed their insights, detailed perspectives, experiences and views on risky riding. Our data limitations however prevent us from being able to report on the specific socio-demographic profile of our participants (other than their Iranian ethnicity), their years of riding experience and their experiences with pervious crashes. Finally, the risk biases and risky behaviors of the motorcyclists in our study are influenced by unmeasured social and other factors, which may be unique to Iranian culture. Therefore, caution should be exercised in the transferability of our findings to other cultures.

Nonetheless, in this paper we have utilized criteria and methodology that enhanced the scientific rigor and credibility of our work while serving to minimize the inherent limitations characteristic of qualitative approaches [36,60]. These include: 1) selection of two different methods of data collection and different sites to verify view points and experiences of the participants (*triangulation*); 2) employing a rigorous data collection protocol that supported saturation of the information collected (*purposive sampling*); 3) using multiple reviewers to ensure adequate presentation and interpretation of the study data. In addition, this is the first study examining risk biases towards and justification for risky riding among motorcyclists living in Iran. In this respect, our findings add to and support the findings from other countries in understanding their road safety behavior [16,61].

## Conclusion

Findings of this qualitative study inform us of four seemingly different categories of riders with different underlying risk biases that justified or facilitated their risky riding behaviors. The categories include; Risk Managers, Risk Utilizers, Risk Calculators, and Risk Takers. The Risk Managers justified risky riding by doubting that it would result in negative outcomes if one is a competent rider. Risk Utilizers justified risky riding as functional and practical; facilitating daily chores and responsibilities. Risk Calculators justified risky riding behaviors that were calculative and believed helped to avoid road crashes. Risk Takers justified risky riding for its thrill and associated peer recognition.

Our findings suggest that a ‘one size fits all’ approach to enhance road safety practice among Iranian motorcyclists may not adequately address the unique characteristics of the subgroups within this population. Nevertheless, our results add support for road safety interventions that aim to find a right match for road safety messages, measures, or incentives and the unique characteristics of groups of risky riders.

### Future directions

Further studies are needed to engage motorcyclists with different risk biases and justifications for risky riding to identify riding incentives or enforcement measures that would motivate and engage them in safe riding practices. Currently in Iran, riders who violate traffic laws, depending on the degree of violation, are subjected to monetary fine, imprisonment, or having their license seized or suspended. Our findings suggest that Risk Takers may be less motivated by the threat of having their motorcycle confiscated since these riders value the motorcycle for seeking pleasure and thrills, while Risk Utilizers who have a more utilitarian view of their motorcycle may have different view of the punishment. Large scale observational studies are needed to report level of readiness to change risky riding of riders with different risk biases to safety riding, and identify what it takes for these riders to adopt safe riding. Such studies should factor in the riders’ perception of current road safety policies and characteristics of the road transport system in Iran [8], and road behaviors of pedestrians and riders of other means of transportation [8,62].

## Competing interests

The authors declare that they have no competing interest.

## Authors’ contributions

FZA was the study main investigator, contributed to the development of research protocol, implementation of the research, analysis of data, and drafted the results of the study. EM was the study co-investigators who coordinated the implementation of the study, participated in data collection, and assisted with data analysis. MB assisted with data interpretation and drafting of the manuscript. SHB assisted with the interpretation of the results, and participated in the drafting, revision, and writing the final draft of the manuscript. DH assisted with the interpretation of the results, revised and assisted with the final draft of the manuscript. All the authors have read and approved the final manuscript.

## Pre-publication history

The pre-publication history for this paper can be accessed here:

http://www.biomedcentral.com/1471-2458/13/865/prepub
